# Does neuropathic low back pain negatively influence work capacity? A cross-sectional study among Hungarian workers

**DOI:** 10.1186/s13690-025-01674-5

**Published:** 2025-07-09

**Authors:** Imre Varadi, Tibor Toth, Eva Fejes, Zsolt Nemeskeri, Miklos Kovacs, Antal Tibold, Rita Nyulas, Gergely Feher

**Affiliations:** 1 Centre for Occupational Medicine, Medical School, University of Pécs, Pecs, 7624 Hungary; 2 Margit Slachta National Social Policy Institute, Budapest, 1142 Hungary; 3 Hospital of Komlo, Clinical Center, University of Pécs, Komlo, 7300 Hungary; 4 Faculty of Cultural Sciences, Education and Regional Development, University of Pécs, Pecs, 7633 Hungary; 5 Baranya County SZC Zipernowsky Károly Technical College, Pécs, 7626 Hungary

**Keywords:** Low back pain, Neuropathic low back pain, Depression, Insomnia, Quality of life, Work Ability Index

## Abstract

Low back pain (LBP) is a leading cause of disability worldwide and neuropathic low back pain (NLBP) represents a subset of LBP that involves nerve injury or dysfunction. This condition has been shown to have significant impacts on physical, psychological, and social well-being. The aim of our study was to investigate the association of NLBP on Work Ability Index (WAI) among postal delivery workers taking many covariates into account. Demographic data, risk factors and concomitant diseases were included into our analysis as well as psychometric questionnaires of burnout (Mini Oldenburg Questionnaire—MOLBI), depression (Beck Depression Inventory—BDI) and insomnia (Athens Insomnia Scale—AIS). Low back pain categories (nociceptive, mixed or neuropathic) were assessed by the painDETECT questionnaire. Working capacity was measured by the Work Ability Index (WAI)*.* This cross-sectional study was conducted between May 2021 and January 2022 among postal delivery workers in Hungary. Overall 1034 responders took part in our survey, 368 males (35.6%) and 666 females (64.4%). Based on the results of the WAI questionnaire, participants could be divided as having poor (3.8%, 39/1034), moderate (18.5%, 191/1034), good (43.8%, 453/1034) and excellent 33,9%, 351/1034) working ability. Due to the low number of participant in the poor WAI group after the setup of a logistic regression analysis we also employed LASSO (Least Absolute Shrinkage and Selection Operator) regression with tenfold cross-validation as well as we applied Firth’s penalized likelihood regression, which reduces small-sample bias in logistic regression. In the final analysis poor WAI was associated with depression (aOR = 2.31, 95% CI: 1.89–2.82, *p* = 0.008), sleep disturbance (aOR = 1.85, 95% CI: 1.42–2.41, *p* = 0.012) and NLBP (aOR = 3.10, 95% CI: 2.95–3.98, *p* = 0.002). Targeted interventions addressing depression and sleep disturbance in individuals with NLBP may help preserve or improve work ability and overall functioning.

**Table:** 

Text box 1. Contributions to literature
– Our study is among the first ones showing the association of reduced working capacity measured by the Work Ability Index (WAI) and neuropathic low back pain (NLBP)
– These workers experience significant impairments in functional performance and physical capabilities, highlighting the necessity for targeted interventions to address these deficits.
– Utilizing assessment tools such as WAI can aid healthcare professionals in measuring the severity of NLBP and its impacts on daily functioning and work ability.

## Introduction

The Work Ability Index (WAI) is an important tool used to evaluate an individual’s capacity to perform work-related tasks, which was developed nearly 30 years ago [1]. It is widely used in both clinical studies and daily routine for assessing work ability and it is widely used by a cluster of healthcare specialists for individual case management, population health trend analysis, health promotion, and for calculating work ability across different target groups [2]. The WAI considers various factors, including physical and mental health, work environment, and personal health history and is commonly used in occupational healthcare systems [3].

Chronic low back pain (CLBP) is one of the most prevalent health issues globally, with a significant impact on both individuals and healthcare systems. CLBP is considered as one of the most devastating pain syndromes with nociceptive and neuropathic components [4]. The rate of neuropathic low back pain (NLBP) fluctuates between 16–55% amongst all CLBP syndromes and is characterized by the presence of nerve damage or dysfunction, which often leads to more complex and persistent symptoms [5]. Recent studies have shown that NLBP tends to be more persistent and harder to treat, leading to significant physical and psychological burden [4, 5]. Patients with neuropathic pain exhibited more severe pain levels detected as well as significantly greater severity of foraminal and spinal stenosis than those with pure nociceptive pain, which underscores the multifactorial etiology of NLBP and stress the need to account for diverse pathological mechanisms beyond neural compression in the assessment and treatment of patients with back and leg pain [6]. For example, specific questionnaires can potentially be utilized as a screening tool to predict opioid response in patients suffering with different types of CLBP [7].

CLBP/NLBP is usually accompanied by several mental issues. Depression is a common comorbidity in individuals with different chronic pain syndromes. Structural and functional magnetic brain imaging studies showed that chronic pain can be associated with alterations in brain chemistry and functioning, which may result in symptoms of depression [8]. Furthermore the psychological distress of persistent pain can lead to sleep disturbances, including severe insomnia. The association is bidirectional as sleep disturbance (insomnia) can be both a consequence of pain and a possible contributing factor to its persistence, creating a nearly unbreakable cycle [9]. Depression and insomnia, especially when combined with NLBP, create a challenging clinical picture that requires a multidisciplinary approach. The presence of depression can worsen the perception of pain, and insomnia can hinder the body’s natural healing processes, both of which further deteriorate the patient’s overall well-being and quality of life [10]. Furthermore, a recent study has highlighted the possible role of the gut microbiome as a shared mechanism linking non-spinal comorbidities—such as gastrointestinal disorders, depression, and rheumatoid arthritis—with NLBP [11].

Burnout is often the result of prolonged exposure to chronic stress, particularly in demanding work environments such as healthcare workers. It can manifest in a variety of ways, including physical exhaustion, a sense of emotional detachment from one’s work, and a perceived lack of personal achievement [12]. The condition can have serious consequences for both personal and professional life, can lead to a cluster of mental and physical issues with a general decline in an individual's health. Burnout and NLBP are two significant health issues which are often considered separate conditions, there is a growing body of research suggesting a complex interaction between mental and physical health [13]. Burnout can exacerbate physical conditions such as NLBP, leading to a vicious cycle of worsening symptoms. Similarly, chronic pain from NLBP can contribute to or trigger burnout, creating a challenging situation for individuals to manage both their physical and mental health. We have recently shown the possible association of burnout and NLBP, which can be mostly triggered by emotional exhaustion [4, 13].

Quality of life (QoL) refers to an individual’s overall well-being, encompassing physical, emotional, and social aspects of health. NLBP, particularly when associated with mental issues and burnout, can severely reduce QoL [14]. Physical limitations due to pain and the inability to perform everyday tasks can lead to social isolation and reduced engagement in recreational activities. Moreover, psychological distress further exacerbates these challenges, creating a significant negative impact on both personal and professional life [4, 13, 14].

For individuals with NLBP, the WAI can provide valuable insights into how the complexity of pain affects their ability to maintain employment and productivity. Reduced work ability can have profound implications on financial stability and overall well-being, making the WAI an essential component of assessing the comprehensive impact of NLBP. Several studies have found that NLBP is linked to greater disability and reduced quality of life; however, these findings were based almost exclusively on assessments using the Oswestry Disability Index [15]. However, studies focusing on the association of workability (or disability) measured by the WAI and NLBP are almost absent [2, 16].

We have previously shown the complexity of burnout and NLBP amongst different populations including postal workers [4, 13, 14]. The aim of our study was to investigate the interrelationships between WAI and NLBP taking many covariates into account such as detailed demographics, risk factors and medical history, mental issues (burnout, depression and insomnia), and QoL including the same population—blue-collar (postal) workers.

## Materials and methods

This study was carried out between 05/2021 and 01/2022 amongst delivery workers of the Hungarian Post recruiting from five counties in Southern-West Hungary. Our study was a cross sectional study in nature applying an online questionnaire with a non-probability sampling method. The study protocol was approved by the Ethics Committee of the University of Pécs as well as the local management of the Hungarian Post Office (reference number: PTE/96773–2/2018). The recruitment process was carried out by the HR Department of the Hungarian Post through the internal mail network. An online link containing our self-completed questionnaire was established during the study period and was sent within their own mail network reaching all postal workers. Participation was anonymous and voluntary; only employees working in the logistics division who were certified as fit for work by the occupational health physician, aged > 18 years, with previously signed online consent forms were allowed to participate in the survey. Individuals deemed partially fit or unfit for work were excluded. Due to the decentralized nature of the recruitment process, exact data regarding the number of questionnaires distributed and the total number of eligible employees are unavailable, which precludes calculation of a precise response rate [4, 14].

*Included demographics* were age, gender, marital status, number of children, years spent with work, number of workplaces and secondary employment.

*Risk factors* contained questions about smoking, alcohol consumption and drug intake. *Medical history* included taking medication regularly, diabetes, hypertension, ischemic heart disease, cerebrovascular disease, peripheral arterial disease, generalized pain (pain involving more than one area of the body) and history of depression.

Low back pain was assessed by the *painDETECT questionnaire* and burnout was measured by the *modified Oldenburg Questionnaire (Mini-OLBI)*. Depression was detected by the *short version of the Beck Depression Inventory* and sleep disturbance was assessed by the *Athens Insomnia Scale* (AIS). Quality of life was surveyed with the *EQ-5D (health-related quality of life)* questionnaire. These questionnaires have already been available and validated in Hungarian language and details were published before [4, 13, 14].

Working capacity was assessed by the *Work Ability Index (WAI)*, which includes a list of diseases and seven occupational health characteristics. The health characters are: a.) current work ability compared to lifetime best ranging from 0–10, b.) work ability in relation to job demands ranging from 2–10, c.) number of currently diagnosed diseases ranging from 1–7, d.), disease estimated work impairment ranging 1–6, e.) being on sick leave in the last 12 months ranging 1–5, f.) own prognosis of work ability in the next 2 years ranging 1–7 and g.) mental resources ranging 1–4 [17]. Based on the sum of the scores mentioned above, individuals can be classified as having excellent (WAI 44–49), good (WAI 37–43), moderate (WAI 28–36), and poor (WAI ~ 27) work abilities. This questionnaire is available and validated in Hungarian language [18].

### Statistical analysis

Data were evaluated as means ± SD (standard deviation), frequencies, and percentages. The chi-square test, the Pearson’s Rank-Order Correlation, distribution ratios and Analysis of variance (ANOVA) were conducted to evaluate differences between study subgroups. Due to the low number of participants in the poor WAI group, the Work Ability Index was dichotomized for the regression analysis: the ‘poor’ category (WAI ≤ 27) was compared to all other categories (moderate to excellent, WAI ≥ 28). Binary logistic regression analysis was used to determine the significance of the included parameters as independent risk factors in the development of NLBP. For all odds ratios, an exact confidence interval (CI) of 95% was constructed in our study. Due to the low number of participant in the poor WAI group after the setup of a binary logistic regression analysis we also employed LASSO (Least Absolute Shrinkage and Selection Operator) regression with tenfold cross-validation as well as we applied Firth’s penalized likelihood regression, which reduces small-sample bias in logistic regression. Statistical analyses were conducted using SPSS version 28.0 (IBM Corp., Armonk, NY, USA) for basic descriptive and inferential statistics, including chi-square tests, ANOVA, and standard logistic regression models. For advanced modeling, including feature selection and bias-reduction techniques, we employed R version 4.3.1 using the glmnet package for LASSO regression and the logistf package for Firth’s penalized likelihood logistic regression. 

## Results

Overall 1034 responders took part in our survey, 368 males (35.6%) and 666 females (64.4%) 43.9% of the examined workers were between 46–55 years of age. 764 participants (73.9%) were married or lived in a relationship. The number of childless people was 236 (22.8%). 58 participants (5.6%) had secondary employment. Vast majority (45.8%) of our study population has been working for 21–40 years.

445 (43.0%) participants were taking medications regularly, 299 (28.9%) were regular smokers, 91 (8.8%) took alcohol and 34 (3.3%) used drugs more or less regularly. 305 (29.5%) participants had a history of hypertension, 185 (17.9%) had musculoskeletal pain. Ischemic heart disease can be detected in 124 (12.0%) workers. 62 (6.0%) participants had diabetes. Detailed demographics has already been published elsewhere [4, 11].

Low back pain occurred in 182 workers (17.6%). Among them 90 workers (49.5%) had nociceptive low back pain (final score: 8–12), 56 workers (30.8%) had unclear or mixed pain (final score 13–18 points) and 36 (19.7%) had neuropathic low back pain (final score 19–38 points).

The prevalence of exhaustion among the study population was 52.8% (546/1034). The mean exhaustion score was 2.3 ± 0.52. 816 workers (78.9%) were disappointed, the mean score was 2.5 ± 0.58. 375 (36.2%) participants had no signs of current depression, 585 (56.6%) currently suffered from mild, 64 (6.2%) from moderate, and 10 (10.0%) from severe depression based on the results of the BDI. Vast majority of the study population (801 participants, 77.5%) had no sleep disturbance), while 17.9% (185/1034) suffered from mild and 4.6% (48/1034) suffered from severe insomnia. Details of the above mentioned results have already been published elsewhere [4, 11].

Based on the results of the WAI questionnaire participants could be divided as having poor (3.8%, 39/1034), moderate (18.5%, 191/1034), good (43.8%, 453/1034) and excellent 33.9%, 351/1034) working ability (Table 1).

**Table 1:** Association between WAI and demographic characteristics among Hungarian postal workers (May 2021–January 2022) (WAI: work ability index, * *p* < 0.05)

(data %. *N* = 1034)	**Poor (*N* = 39)**	**Moderate (*N* = 191)**	**Good (*N* = 453)**	**Excellent (*N* = 351)**	**Total**	**p**
**Gender X**^**2**^** (1) = 2.225**						
Male	17 (43.6%)	91 (47.6%)	143 (31.6%)	117 (33.3%)	368	0.311
Female	22 (56.4%)	100 (52.4%)	310 (58.4%)	234 (66.7%)	666	0.205
**Age X**^**2**^** (5) = 3.610**						
18–25 years	1 (2.5%)	4 (2.1%)	10 (2.2%)	26 (7.4%)	41	0.509
26–35 years	4 (10.3%)	19 (10.0%)	51 (11.3%)	64 (18.2%)	138	0.437
36–45 years	7 (17.9%)	40 (20.9%)	95 (21.0%)	101 (28.8%)	243	0.308
46–55 years	14 (35.9%)	87 (45.6%)	228 (50.3%)	125 (35.6%)	454	0.969
56–62 years	9 (23.1%)	31 (16.2%)	69 (15.2%)	33 (9.4%)	142	0.033*
> 62 years	4 (10.3%)	10 (5.2%)	0 (0%)	2 (0.6%)	16	0.607
**Marital status X**^**2**^** (3) = 0.667**						
Single	3 (7.7%)	19 (9.9%)	58 (12.8%)	53 (15.1%)	133	0.881
Relationship	8 (20.5%)	41 (21.5%)	60 (13.2%)	68 (19.4%)	177	0.474
Married	19 (48.7%)	103 (53.9%)	269 (59.4%)	196 (55.8%)	587	0.908
Divorced / widow	9 (23.1%)	28 (14.7%)	66 (14.6%)	34 (9.7%)	137	0.562
**Number of children X**^**2**^** (3) = 5.998**						
no child	10 (25.6%)	45 (23.6%)	89 (19.6%)	92 (26.2%)	236	0.112
1 child	10 (25.6%)	57 (29.8%)	119 (26.3%)	83 (23.6%)	269	0.243
2 children	13 (33.4%)	65 (34.0%)	182 (40.2%)	125 (35.6%)	385	0.075
≥ 3 children	6 (15.4%)	24 (12.6%)	63 (13.9%)	51 (14.5%)	144	0.344
**Number of workplace X**^**2**^** (4) = 7.123**						
1st	8 (20.5%)	35 (18.3%)	211 (46.6%)	160 (45.6%)	414	0.279
2nd	9 (23.1%)	30 (15.7%)	98 (21.6%)	59 (16.8%)	196	0.477
3rd	4 (10.2%)	34 (17.8%)	78 (17.2%)	67 (19.1%)	183	0.380
4th	9 (23.1%)	22 (11.5%)	44 (9.7%)	19 (5.4%)	94	0.013*
5th or more than 5th	9 (23.1%)	70 (36.7%)	22 (4.9%)	46 (13.1%)	147	0.023*
**Years spent with work X**^**2**^** (6) = 4.831**						
1–12 months	1 (2.5%)	7 (3.7%)	19 (4.2%)	18 (5.1%)	45	0.950
1–5 years	4 (10.3%)	35 (18.3%)	77 (17.0%)	94 (26.8%)	210	0.882
6–10 years	3 (7.7%)	17 (8.9%)	47 (10.4%)	40 (11.4%)	107	0.936
11–20 years	7 (17.9%)	29 (15.2%)	86 (19.0%)	59 (16.8%)	181	0.827
21–30 years	13 (33.4%)	52 (27.2%)	123 (27.2%)	77 (21.9%)	265	0.807
31–40 years	7 (17.9%)	47 (24.6%)	96 (21.2%)	56 (16.0%)	206	0.948
more than 40 years	4 (10.3%)	4 (2.1%)	5 (1.1%)	7 (2.0%)	20	0.907

Poor WAI was associated being 56–62 years old (23.1% vs 9.4%, *p* = 0.033) and having four (23.1% vs 5.4%, *p* = 0.013) and five or more workplaces (23.1% vs 13.1%, *p* = 0.023) (Table 1).

There was a significant association between poor WAI and taking medication regularly (76.9% vs 19.9%, *p* = 0.008), drinking alcohol more or less regularly (20.5% vs 5.4%, *p* < 0.001) as well as with the history of musculoskeletal pain (64.1% vs 1.7%, *p* < 0.001), hypertension (58.9% vs 11.1%, *p* = 0.011) and previous spinal surgery (5.1% vs 0.9%, *p* = 0.011) (Table 2).

**Table 2:** Association of WAI, risk factors and medical history among Hungarian postal workers (May 2021–January 2022) (WAI: work ability index, * *p* < 0.05, **p < 0.001)

(data %. *N* = 1034)	**Poor (*N* = 39)**	**Moderate (*N* = 191)**	**Good (*N* = 453)**	**Excellent (*N* = 351)**	**Total**	**p**
**Comorbidity**						
taking medication regularly X^2^ (1) = 0.967	30 (76.9%)	116 (60.7%)	229 (50.6%)	70 (19.9%)	445	0.008*
smoker X^2^ (1) = 0.385	15 (38.5%)	67/191 (35.1%)	126/453 (27.8%)	91 (25.9%)	299	0.261
taking alcohol X^2^ (1) = 12.380	8 (20.5%)	32 (16.8%)	32 (7.1%)	19 (5.4%)	91	< 0.001**
taking drugs X^2^ (1) = 0.032	1 (2.5%)	11 (5.8%)	14 (3.1%)	8 (2.3%)	34	0.858
diabetes X^2^ (1) = 0.086	6 (15.4%)	13 (6.8%)	36 (7.9%)	7 (2.0%)	62	0.082
hypertension X^2^(1) = 0.551	23 (58.9%)	82 (42.9%)	161 (35.5%)	39 (11.1%)	305	0.011*
ischemic heart disease X^2^ (1) = 0.156	14 (35.9%)	40 (20.9%)	61 (13.5%)	9 (2.6%)	124	0.107
musculoskeletal pain X^2^ (1) = 0.224	25 (64.1%)	71 (37.2%)	83 (18.3%)	6 (1.7%)	185	< 0.001**
stroke X^2^ (1) = 0.013	2 (5.1%)	7 (3.7%)	7 (1.5%)	2 (0.6%)	18	0.338
vascular disease X^2^(1) = 0.051	8 (20.5%)	20 (10.5%)	28 (6.2%)	2 (0.6%)	58	0.182
cancer X^2^ (1) = 0.030	2 (5.1%)	7 (3.7%)	8 (1.8%)	7 (2.0%)	24	0.623
mental disorder X^2^(1) = 0.044	9 (23.1%)	20 (10.5%)	21 (4.6%)	1 (0.3%)	51	0.099
spinal surgery X^2^ (1) = 49.554	2 (5.1%)	5/ (2.6%)	7 (1.5%)	3 (0.9%)	20	< 0.001**

There was a significant association between Work Ability Index and mild or moderate depression (64.1% vs 37.3%, *p* = 0.011, 28.3% vs 1.1%, *p* = 0.006), sleep disturbance (51.3% vs 4.8%, *p* = 0.004, 30.8% vs 0.3% *p* = 0.031) and unclear and neuropathic pain (17.9% vs 1.4%, *p* = 0.048, 15.4% vs 3.8%, *p* = 0.012) (Table 3).

**Table 3:** Association of WAI with mental issues and neuropathic low back pain among Hungarian postal workers (May 2021–January 2022) (WAI: work ability index, * *p* < 0.05)

(data %. *N* = 1034)	**Poor (*N* = 39)**		**Moderate (*N* = 191)**	**Good (*N* = 453)**	**Excellent (*N* = 351)**	**Total**	**p**
**Depression X**^**2**^** (3) = 2.271**							
normal		2 (5.1%)	24 (12.6%)	136 (30.0%)	213 (60.7%)	375	0.265
mild		25 (64.1%)	136 (71.2%)	293 (64.7%)	131 (37.3%)	585	0.011*
moderate		11 (28.3%)	25 (13.1%)	24 (5.3%)	4 (1.1%)	64	0.006*
severe		1 (2.5%)	6 (3.1%)	0 (0%)	3 (0.9%)	10	0.482
**Burnout** X^2^(3) = 4.289		15 (38.5%)	62 (32.5%)	213 (47.0%)	235 (67.0%)	525	0.093
exhaustion		16 (41.0%)	69 (36.1%)	226 (49.9%)	235 (66.1%)	546	0.737
disappointment		26 (66.7%)	134 (70.2%)	358 (79.0%)	298 (84.9%)	816	0.139
**Sleep disturbance X**^**2**^** (2) = 0.269**							
no		7 (17.9%)	105 (55.0%)	356 (78.6%)	333 (94.9%)	801	0.520
present		20 (51.3%)	64 (33.5%)	84 (18.5%)	17 (4.8%)	185	0.004*
severe		12 (30.8%)	22 (11.5%)	13 (2.9%)	11 (0.3%)	48	0.031
**Neuropathic pain X**^**2**^** (2) = 5.317**							
nociceptive		4 (10.3%)	17 (8.9%)	39 (8.6%)	30 (8.5%)	90	0.702
unclear pain		7 (17.9%)	24 (12.6%)	20 (4.4%)	5 (1.4%)	56	0.048*
neuropathic pain		6 (15.4%)	13 (6.8%)	17 (3.8%)	0 (0%)	36	0.012*

In the logistic regression analysis we have included those parameters found to be significantly associated with poor WAI in an univariate analysis (age, number of workplaces, sleep disturbance, depression, taking medication regularly, taking alcohol, previous spinal surgery, hypertension, history of musculoskeletal pain, depression, insomnia and neuropathic pain). The model explained 70.2% (Nagelkerke R^2^) of variance in work ability (correctly classified 98.9% of cases). There was significant association between poor WAI and depression (aOR = 2.018, *p* = 0.012, CI 95% 1.036–2.131), sleep disturbance (aOR = 1.894, *p* = 0.006, CI 95% 1.646 to 2.183), taking alcohol (aOR = 1.573, *p* = 0.001 CI 95% 1.390 to 1.806), hypertension (AOR = 1.493, *p* = 0.034, CI 95% 1.030 to 1.950), neuropathic pain (aOR = 3.421, *p* = 0.001 CI 95% 3.189 to 4.011) and history of musculoskeletal pain (aOR = 1.486, *p* = 0.017, CI 95% 1.208 to 1.778), but it should be treated with caution due to the relatively low Nagelkerke *R*^*2*^ value and low number of participants in the poor WAI group, which raises concerns about the validity of these results (Table 4).

**Table 4:** Predictors of poor work ability (WAI ≤ 27) in binary logistic regression among Hungarian postal workers (*n* = 1034) (WAI: work ability index, * *p* < 0.05)

*N* = 1034	aOR	CI 95%
age (> 56)	0.360	0.337—0.379
number of workplaces	0.210	0.107—0.364
depression	2.018*	1.036—2.131
sleep disturbance	1.894*	1.646—2.283
medical use	0.247	0.141—0.426
alcohol consumption	1.573*	1.390—1.806
spinal surgery	0.798	0.579—0.985
musculoskeletal pain	1.486*	1.208—1.778
hypertension	1.493*	1.030—1.950
neuropathic pain	3.421*	3.189—4.01

To address the risk of overfitting due to the limited number of events (*n* = 39 in the poor WAI group) relative to the initial 12 candidate factors, we employed LASSO (Least Absolute Shrinkage and Selection Operator) regression with tenfold cross-validation. This method penalizes non-informative predictors, retaining only those with non-zero coefficients. From the initial set, three variables were selected: depression, sleep disturbance, and neuropathic pain. To mitigate sparse data bias (events per variable [EPV] = 3.25), we applied Firth’s penalized likelihood regression, which reduces small-sample bias in logistic regression.

The final model based on Firth’s penalized likelihood logistic regression including only the LASSO-selected predictors (depression, sleep disturbance, and neuropathic pain) is presented in Table 5. The final model demonstrated good discrimination, with an AUC-ROC of 0.78 (95% CI: 0.72–0.84). Sensitivity and specificity were 82% and 74%, respectively, at a 0.5 probability threshold. Calibration, assessed via the Hosmer–Lemeshow test, indicated no significant deviation between predicted and observed probabilities (*p* = 0.21). Poor WAI was associated with depression (AOR = 2.31, 95% CI: 1.89—2.82, *p* = 0.008), sleep disturbance (aOR = 1.85, 95% CI: 1.42—2.41, *p* = 0.012) and NLBP (aOR = 3.10, 95% CI: 2.95—3.98, *p* = 0.002) (not shown).

**Table 5:** Final model using Firth logistic regression with LASSO-selected predictors among Hungarian postal workers (May 2021–January 2022) (* *p* < 0.05)

Predictor 95%CI	**Adjusted OR**	**CI 95%**
Depression	2.31*	1.89 – 2.82
Sleep disturbance	1.85*	1.42 – 2.41
Neuropathic pain	3.10*	2.95 – 3.98

To evaluate robustness, we excluded participants with *mixed pain* (*n* = 318, 30.8%), as these cases could obscure the true association between neuropathic pain and WAI. In this subgroup, neuropathic pain exhibited a stronger effect (aOR = 3.45, 95% CI: 3.12—4.21, *p* < 0.001), and model discrimination improved (AUC = 0.81, 95% CI: 0.75—0.87). Sensitivity and specificity increased to 85% and 77%, respectively, suggesting that mixed pain cases diluted the neuropathic pain effect in the primary analysis.

Internal validation via bootstrapping (1.000 replicates) confirmed stable estimates, with minimal optimism in AUC (corrected AUC = 0.76). Despite improvements, the small event size (*n* = 39) and reliance on painDETECT scores without clinical confirmation remain limitations. However, LASSO and Firth correction enhanced reliability, increasing the effective EPV to 13 (39 events/3 predictors).

## Discussion

This study investigated the possible association between workability and NLBP taking many covariates into account recruiting a relatively large number of “blue-collar” workers (workers of the Hungarian Post engaged in physical or manual labor such as sorting, handling, and delivering mail and parcels). Nevertheless, focusing on this occupational group is justified by previous research indicating that blue-collar workers are disproportionately affected by work-related musculoskeletal disorders and suffer from lower work ability scores compared to other professions [2, 11, 15]. Our study contributes valuable insights into a vulnerable and understudied segment of the workforce, underscoring the need for tailored interventions and occupational health surveillance in similar labor-intensive settings.

Chronic low back pain is considered as a complex biopsychosocial problem, consisting of a combination of physical (risk factors and diseases), mental (insomnia, depression etc.), social (education, income etc.), and workplace-related factors (work ability, balance of efforts and rewards etc.) [3]. Furthermore, it is a composite pain syndrome containing both (alone or mixed) nociceptive and neuropathic pain elements, the rate of neuropathic pain varies between 16–55% [19]. CLBP is one of the most important disabling health problems, with consistently elevated prevalence worldwide [20]. CLBP currently is the leading cause of disability and absenteeism in the developed world with essential consequences on the individuals’ work, private life, health and social activities [20, 21]. The etiology is multifactorial as seen above, therefore the appropriate intervention is challenging, especially for patients with pure or mixed neuropathic pain [22]. Physical work, including manual work, stooped and prolonged postures and strenuous physical labor may be the most important predecessors of NLBP [23, 24].

Disability is a commonly reported and recognized outcome of CLBP by a cluster of previously validated and widely used patient-reported outcome measures (PROMs) [25]. Work ability is a cornerstone of individuals’ well-being, mental and physical health [2]. The WAI is a widely used tool in occupational medicine for assessing current work ability, but association of chronic pain syndromes with WAI are almost lacking. Prior studies suggested the negative impact on functional status and work ability of CLBP among workers, but studies focusing on the complex association of NLBP and WAI are absent or sparse [2, 16].

In our study a significant proportion of our participants (~ 20%) had poor or moderate WAI, which is comparable to the data of healthcare professionals [26]. Poor WAI was more common amongst participants aged > 56 years. This study similarly to previous studies also confirmed the age-related decrease in work ability, however, it lost its significance in a multivariate analysis and results should be treated with caution due to the low number of participants in the poor WAI group [27, 28].

Having 4 ≥ workplaces was also more common in individuals with poor WAI. The association can be bidirectional as having poor working capacity can be associated with job loss and job satisfaction is negatively correlated with WAI (e.g. dissatisfaction can lead to poor work ability) [2]. On the other hand, this association could not be confirmed by a multivariate analysis and methodological issues also occur as mentioned before.

Taking medications regularly was also significantly associated with poor WAI, but only in an univariate analysis. Regular medication intake probably reflects the presence of one or more chronic diseases, which may result in poorer working capacity as was previously shown in a Finnish survey [29]. Hypertension (as a chronic disease) was also significantly more common in participants with poor WAI in both uni- and multivariate analysis. Cardiovascular diseases are sharply associated with reduced working capacity and hypertension as their predecessor is also associated with the phenomenon, although the statistical significance is slightly weaker [29, 30]. Alcohol consumption is also known to be associated with reduced working capacity, and previous studies also showed its negative role on disability retirement and absenteeism due to depressive disorders, which has also been confirmed by our results, although it should be treated with caution [31, 32].

Prior spinal surgical treatment was also significantly more common amongst those with poor WAI. Having an unnecessary surgical evaluation (e.g. without any warning signs) can exacerbate pre-existing symptoms or result in new complaints by inducing spinal pathologies and increased anxiety [33].

History of musculoskeletal pain as well as the presence of NLBP were also associated with poor WAI in both uni- and multivariate analysis. Chronic or neuropathic pain, if not properly managed, can be a progressive devastating disease for the individual and decline in health status is a significant factor of reduced work capacity [24]. In previous studies musculoskeletal disorders exerted the strongest negative effect on working ability [29, 30]. However, apart from neuropathic pain the above mentioned results were not included in the restricted analysis, and the sensitivity and applicability of the applied model also raise concerns; therefore, these findings should be interpreted with caution.

Mental issues (depression and insomnia) were also more common in the poor WAI group in both uni- and multivariate analysis including the restricted forms of statistics (see below). Chronic/musculoskeletal pain is usually complicated by mental issues such as insomnia, depression burdening both the individual and the society. Poor WAI can be the consequence of pain and its psychiatric complications or inversely, pre-existing mental issues can cause a significant decline in WAI [34, 35].

To address the risk of overfitting due to the limited number of events relative to the initial candidate factors, we employed LASSO (Least Absolute Shrinkage and Selection Operator) regression with 10-fold cross-validation. From the initial set, three variables were selected: depression, sleep disturbance, and neuropathic pain. We also applied Firth’s penalized likelihood regression, which reduced small-sample bias in logistic regression. The final model demonstrated good discrimination. Poor WAI was associated with depression, sleep disturbance and neuropathic pain, which underlines the association of NLBP and mental issues in the development of reduced workability. Mixed pain (containing both nociceptive and neuropathic elements) was also associated with poor workability in a univariate analysis, but it lost its significance in both multivariate analysis. If we excluded those with mixed pain, neuropathic pain exhibited a stronger effect, and model discrimination improved suggesting a diluting role of this pain condition.

Prior studies suggested the negative impact of CLBP on working capacity, but research focusing on the association of NLBP and WAI is poorly investigated/lacking [3, 36]. Our study is among the first ones the first showing the negative impact of NLBP on WAI, the association was confirmed in both uni- and multivariate analysis as well as restricted multivariate analysis. Comparing to nociceptive pain, neuropathic pain or pain with neuropathic elements are typically more intense, persistent, and require other treatment strategies (as being usually unresponsive to conventional therapies e.g. pain killers) than non-neuropathic pain, leading to greater functional impairment. If it is not properly managed, it is associated with reduced work productivity as well as higher risk of work absence and disability claims [5, 37]. Furthermore, there is a strong link between NLBP and mental issues such as depression and sleep disturbances (which were also strongly associated with poor WAI based on our results including restricted analysis) further diminish work capacity [38]. Patients with neuropathic low back-related leg pain often face challenges in returning to work due to persistent symptoms as well as may require ergonomic modifications, flexible work schedules, or changes in job roles to remain productive [13, 39].

### Strengths and limitations

**Fig. 1:**
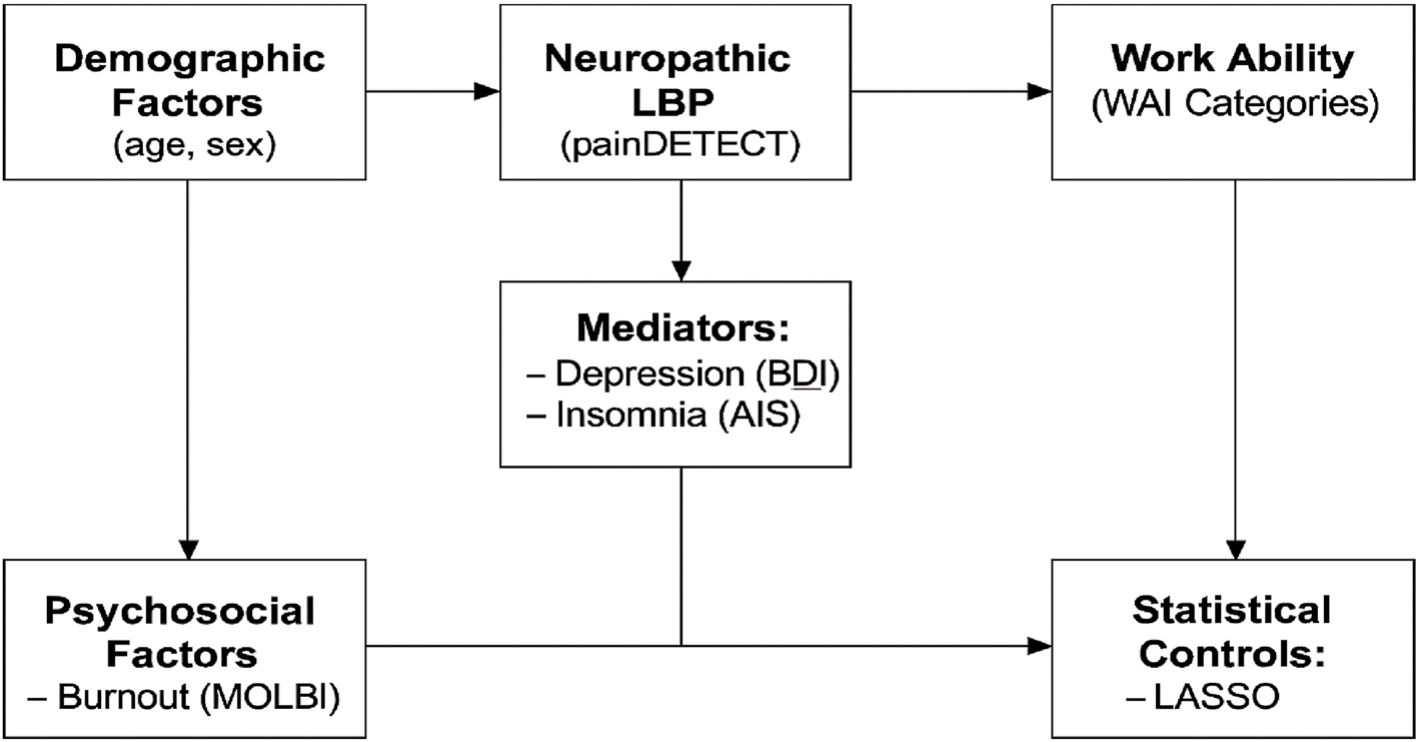
Conceptual framework illustrating the hypothesized relationships between neuropathic pain, mental health conditions, and work ability among Hungarian postal workers (May 2021–January 2022)

This study has several strengths and limitations that must be considered. One of the main strengths is the relatively large sample size (*N* = 1034), which provides sufficient statistical power and improves the reliability of the multivariable regression analyses. The use of validated and widely applied self-report instruments in Hungarian — including the Work Ability Index (WAI), painDETECT, the Beck Depression Inventory (BDI), the Athens Insomnia Scale (AIS), and the EQ-5D — enhances the credibility and comparability of the findings. Another important strength is the focus on a real-world, high-risk occupational population: blue-collar postal workers, who are often underrepresented in occupational health studies despite being disproportionately affected by musculoskeletal disorders. The application of advanced statistical techniques — namely LASSO regression for variable selection and Firth’s penalized likelihood logistic regression to reduce small-sample bias — further supports the robustness of the results. Internal validation via bootstrapping was also performed to ensure the stability of the final model. Finally, the study includes a conceptual framework (Fig. 1), which graphically illustrates the hypothesized associations between neuropathic pain, psychological comorbidities, and reduced work ability. It was an online epidemiological survey; therefore, there was no physical examination, or detailed medical history with regards to the duration of symptoms and previous patient records were also not available, which introduces a potential selection bias and limits generalizability. Additionally, given the online format of the survey, biases such as the healthy worker effect and underrepresentation of severely affected individuals must be considered. The study sample was restricted to postal workers in a single country and recruited using a non-random, convenience sampling method. As such, the generalizability of the results to other occupational groups or countries is limited. Additionally, we did not collect data on whether participants were receiving workers’ compensation benefits as well as key workplace confounders (e.g., job satisfaction, workload), which could potentially influence self-reported symptoms, motivation to return to work, or perceived work ability. Additionally, we did not collect data on workers’ compensation status, which has been shown in prior literature to influence pain outcomes and return-to-work trajectories. The statistical model we applied should be interpreted with caution. Although technically sound—effectively addressing overfitting and small sample size through the use of LASSO, Firth correction, and bootstrapping—the results can be considered reliable but have limited generalizability. Caution is particularly warranted due to the low number of events and the fact that neuropathic pain was assessed using the painDETECT questionnaire, which provides a screening-based rather than a clinically confirmed diagnosis. Future research should also incorporate detailed workplace assessments—including ergonomic evaluations and subjective job satisfaction measures—to improve model accuracy and better delineate the mechanisms linking NLBP and work capacity.

In conclusion, our study is among the first ones showing that NLBP is associated with reduced work ability due to its chronic and severe nature. These workers experience significant impairments in functional performance and physical capabilities, highlighting the necessity for targeted interventions to address these deficits. Utilizing assessment tools such as the Work Ability Index can aid healthcare professionals in measuring the severity of NLBP and its impacts on daily functioning and work ability. Prior to any surgical consideration, patients should undergo comprehensive evaluation by a team comprising physiotherapists, occupational health experts, and spine specialists. Initial management should focus on conservative measures such as physiotherapy, workplace ergonomic modifications, psychological support, and interventions targeting sleep disturbances.

For occupational physicians, the early detection of individuals with overlapping physical and mental health challenges is critical for preserving work capacity. Multidisciplinary rehabilitation programs that simultaneously address neuropathic pain and associated conditions—such as depression and insomnia—may reduce the need for surgical intervention and help mitigate long-term disability. Surgical referral should be reserved for cases unresponsive to conservative therapy, and should ideally be based on clinical verification of neuropathic pathology rather than relying solely on screening tools. To illustrate the hypothesized interactions between neuropathic pain, psychological comorbidities, and reduced work ability, we present a conceptual framework in Fig. 1.

## Data Availability

No datasets were generated or analysed during the current study.
